# 

**Published:** 1921-10

**Authors:** 


### EDITORIAL NOTICE

***The Editor of "The Hospital and Health Review" will be pleased to consider contributions for publication in its columns.***

***The name and address of the writer, not necessarily for publication, should accompany all communications.***

***A stamped and addressed envelope should be enclosed in any case where the writer wishes for the return of an article or desires to correct his own proofs.***

***The Editor will be pleased to receive criticisms and comments from the Readers of this paper.***

***All editorial communications should be addressed to The Editor, "The Hospital and Health Review," 28/9 SouthamptonSt., Strand, London, W.C. 2.***

### A Leaflet Competition

*We offer **Three Guineas** for the best new and original leaflet of not more than 300 words on*

**"The Care of the Teeth"**

The Leaflet must be suitable for circulation in the Public Elementary Schools and among the Scholars of the Secondary Schools. The result of this Competition will be announced and the successful Leaflet will be printed in the November number of "The Hospital and Health Review." We reserve the right to republish any of the Leaflets submitted to us.

The Editor hopes that all sorts and conditions of people, not only Medical Officers of Health and Dental Surgeons, will take part in this Competition. The Americans have told us that "we dig our graves with our teeth," and above all other "minor ailments" at the present time the care of the teeth is the most important.

### RULES AND REGULATIONS.

1. This competition is open to all.
2. Leaflets must be written or typed on unprinted paper on one side of the page only.
3. Competitors must employ a pseudonym.
4. Leaflets must be received by The Editor at "The Hospital and Health Review," 28 Southampton Street, Strand, W.C. 2, by **the first post on November 3rd, 1921.** Envelopes must be marked in the top left-hand corner with the word "Leaflet."
5. The Editor's decision is final.

### CORRESPONDENCE.

[Correspondence on all subjects is invited, but we cannot in any way be responsible for the opinions expressed by our correspondents, who must give their name and address as a guarantee of good faith, but not necessarily for publication. Correspondents are reminded that brevity of style and conciseness of statement greatly facilitate early insertion.]

### Hospital Representation on the King's Fund.

*To the Editor of* THE HOSPITAL AND HEALTH REVIEW.

### APPOINTMENTS.

### Public Health.

DR. B. C. STEVENS, M.D., M.S.Durh., to be medical officer of health for Grimsby.

Mr. D. I. Walker, M.B., Ch.B.Aberd, to be assistant school medical officer to the Dundee Education Authority.

Miss Marion Draper, M.B., Ch.B.Leeds, to be assistant medical officer of health for Swindon.

Messrs. S. P. Hosegood, L.R.C.P.Lond., M.R.C.S., J. F. D. Willoughby, L.R.C.P.Lond., M.R.C.S., and J. A. Mathers, L.R.C.P.&S.Edin., to be certifying surgeons under the Factory and Workshops Acts.

### Institutional.

Miss Lucy Allen to be matron to the Frenchay Park Children's Sanatorium, Bristol.

Miss Jane Watson to be matron to Bury Infirmary.

Miss Clara Allen to be second assistant matron to the Withington Institution, West Didsbury, Manchester.

Miss Sarah Ann Cross to be matron to the Woolwich Home for Ailing Babies.

Miss E. A. Woodward to be matron to St. Luke's Hospital, Halifax.

Miss Emily Miller to be assistant matron to the Wingrove Hospital, Newcastle-on-Tyne.

Miss Dorothy Swift to be matron to the Lloyd Hospital, Bridlington.

Miss E. C. Stewart to be matron to the Upperton Sanatorium, Lanarkshire.

### MANAGERIAL NOTICES.

**All letters on business matters must be addressed to The Manager, "The Hospital and Health Review," 28 and 29 Southampton Street, London, W.C. 2.**

RATES FOR SUBSCRIPTION (payable in advance).

| | | |
|---|---|---|
| Three Months (including Postage) | ... ... | 2s. 0d. |
| Six Months do. | ... ... | 3s. 9d. |
| Twelve Months do. | ... ... | 7s. 6d. |

Subscriptions (which may commence at any time), are payable in advance. Cheques and Post-Office Orders (crossed London County and Westminster Bank, Covent Garden Branch) should be made payable to the Manager of THE HOSPITAL AND HEALTH REVIEW.

**It is especially requested that remittances be made by Cheque or Postal Order and *NOT* Stamps.**

***Advertisements.*** Scale of charges and particulars of special positions may be obtained on application to the Manager.

